# The Effect of Hole Geometry on the Nonlinear Nanomechanics of *γ*-Graphyne Structures: A Finite Element Analysis

**DOI:** 10.3390/ijms241914524

**Published:** 2023-09-25

**Authors:** Stelios K. Georgantzinos, Sotirios G. Siampanis, Nikolaos Rogkas, Vasilios Spitas

**Affiliations:** 1 Laboratory for Advanced Materials, Structures and Digitalization, Department of Aerospace Science and Technology, National and Kapodistrian University of Athens, 34400 Psachna, Greece; ssiampanis@uoa.gr; 2 General Department, National and Kapodistrian University of Athens, 34400 Psachna, Greece; 3 Machine Design Laboratory, School of Mechanical Engineering, National Technical University of Athens, 15772 Athens, Greece; nrogkas@mail.ntua.gr (N.R.); vspitas@central.ntua.gr (V.S.)

**Keywords:** *γ*-graphyne structures, hole, nonlinear finite element analysis, mechanical properties

## Abstract

Graphyne is a material that has unique mechanical properties, but little is known about how these properties change when the material has holes. In this work, the effect of hole geometry, considering circular, triangle, and rhombus hole configurations, on the mechanical nonlinear response of *γ*-graphyne structures is studied. Graphyne, graphdiyne, graphyne-3, and graphyne-4 structures are under investigation. An efficient nonlinear finite element analysis (FEA) method is adequately implemented under large deformations for this purpose. The study varied the size and shape of the holes to understand how these changes affect the nanostructure’s mechanical response. The results indicate that the hole geometry significantly impacts the mechanical nonlinear response of *γ*-graphyne structures. The holes’ size and shape affect the structures’ elastic behavior, deformation, and strength. The findings can be used to optimize the design of *γ*-graphyne structures for specific mechanical applications. The study highlights the importance of considering the hole geometries in the design and fabrication of these materials.

## 1. Introduction

The introduction of monolayer graphene in 2004 was a scientific breakthrough in the field of materials due to its combination of superior mechanical, electromagnetic, optical, and thermal properties [1,2]. In recent years, efforts have been made to discover materials and structures at the nanoscale that are akin to graphene or possess superior physical and structural attributes compared to graphene. Among the most promising recently identified graphene-like nanomaterials is the family of graphyne materials, initially examined theoretically by Balaban and colleagues [3], and subsequently by Narita and Nagai through first-principles analysis [4]. Graphyne represents a novel category of two-dimensional (2D) substances that have recently garnered interest owing to their distinct physical and chemical characteristics. Comprising sp- and sp^2^-bonded carbon atoms, these substances exhibit a honeycomb configuration akin to graphene, but with extra atoms or functional groups affixed to the carbon atoms. The presence of these additional atoms or groups gives rise to a wide range of properties, including enhanced electrical conductivity, mechanical strength, and chemical stability [5,6,7].

Despite the successful synthesis of graphdiyne by Li et al. in 2010 [8], there are limited experimental data available on the mechanical properties of graphyne structures in the literature. According to Ivanovskii [9], various types of graphyne and graphdiyne were thoroughly investigated for basic structural and mechanical properties, which were determined by experiments or evaluated by ab initio theory. Additionally, as reported by Zhang et al. [10], the Raman spectra of graphyne and graphdiyne were examined in a systematic manner, and the behavior of these materials under mechanical stress and strain was also investigated. A study by Xiao et al. [11] used atomic force microscopy experiments to determine the thermomechanical properties of graphdiyne films. 

The effective design of advanced applications using nanostructures requires a thorough understanding of their properties and an ability to predict them using reliable theoretical or computational tools. Numerous molecular dynamics (MD) simulations and ab initio studies have been carried out on structures like graphene, carbon nanotubes, and fullerenes to comprehend a broad spectrum of properties [12,13,14] and behaviors [15,16,17]. Additionally, the finite element method has been used to provide a more comprehensive approach to the computational modeling of nanostructures. With regard to nanostructures, several forms of finite elements, such as beams [18,19,20], spring-like elements [21,22,23], shells [24,25], and bar elements [26,27], have been formulated to analyze the mechanical performance of these materials. 

Atomistic finite element models simulate interatomic interactions of atoms using appropriate finite element formulations. Couto and Silvestre [28] introduced an FEA modeling approach to estimate the elastic behavior of graphyne. They conducted uniaxial and biaxial tensile simulations using computational models of zigzag and armchair configurations and compared their results with equivalent molecular dynamics and density functional theory models, finding that they were in good agreement. Lee et al. [29] applied an atomistic FE model to analyze the sensitivity of various graphyne-based resonators under different boundary conditions and size configurations. They compared the results with those of graphene-based resonators and concluded that graphyne derives higher sensitivity. Georgantzinos et al. [30,31] investigated the elastic and elastoplastic mechanical properties of *γ*-graphyne family structures utilizing FE analysis. They conducted various parametric studies, concentrating on the size-dependency of the sheet structure on the mechanical properties, and generalized their results using regression models. Galhofo and Silvestre [32] derived stress–strain curves to examine both the monotonic and hysteretic behavior of γ-graphyne sheets using the same geometric configurations as in [28]. Siampanis et al. [33] examined the stress–strain behavior of graphyne structures under shear loading and obtained higher shear strength and stiffness compared to other structures of this family. 

The available studies on the mechanical properties of graphyne structures by numerical methods assume ideal sheet structures without any defects or holes. However, in the case of graphene, a particular interest in the modeling of the structure lies in the assessment of the influence of nano-holes on the mechanical behavior. This is in line with the investigation of nanoporous graphene or graphene sheet with holes that have been studied due to its good properties for gas absorption [34], water desalination [35], energy storage [36,37], and nanoelectronics applications [38]. Genoese et al. [39] introduced a new interatomic potential, the Dreading potential, in which they added damping functions, to investigate the in-plane and out-plane tensile behavior of monolayer graphene sheets. In their study, they examined the effect of the diameter of a hole located in the middle of the sheet on the stress and strain distributions. Another study on the effect of holes on graphene sheets can be found in [40], where Muraru et al. developed a new software tool to generate graphene-based molecular models. Yoon et al. [41] examined the mechanical resonance properties of porous graphene sheets computationally and experimentally. However, graphyne structures fall behind in terms of published papers regarding the role of holes and their effect on mechanical properties. Recently, Lee et al. [42] used an atomistic FE model to examine the effect of holes on Young’s moduli of porous γ-graphyne sheets under tensile loading, concluding that an increase in porosity decreases Young’s modulus. 

The presence of holes in a graphyne structure can play a practical role in several areas such as electronics, energy storage, and catalysis. In electronics, the holes can be used to create electronic devices with high electron mobility, such as field-effect transistors. In energy storage, graphyne with holes can be used as a high-capacity electrode material in lithium-ion batteries. The holes can also be used as active sites for catalytic reactions in chemical synthesis, such as hydrogenation reactions. The size and shape of the holes can also be used to control the behavior of the graphyne, such as its strength, electronic conductivity, and chemical reactivity. Additionally, by controlling the size and shape of the holes, it may be possible to create new types of graphyne structures with unique properties that can be used in a variety of applications. As a recent addition to the family of carbon allotropes, graphyne structures have not been extensively studied in comparison to other carbon nanostructures. In this study, we investigate the effect of hole geometry on the mechanical nonlinear response of γ-graphyne structures, using an efficient nonlinear finite element method (FEM). Specifically, we examine the mechanical properties of graphyne, graphdiyne, graphyne-3, and graphyne-4 structures with circular, triangle, and rhombus hole configurations. The implementation of the FEA model is conducted under large deformations, and it is an atomistic spring-based finite element model in which a unique spring is used to simulate the interatomic interactions between carbon atoms.

## 2. Computational Model

### 2.1. Geometric Characteristics

**Figure 1:**
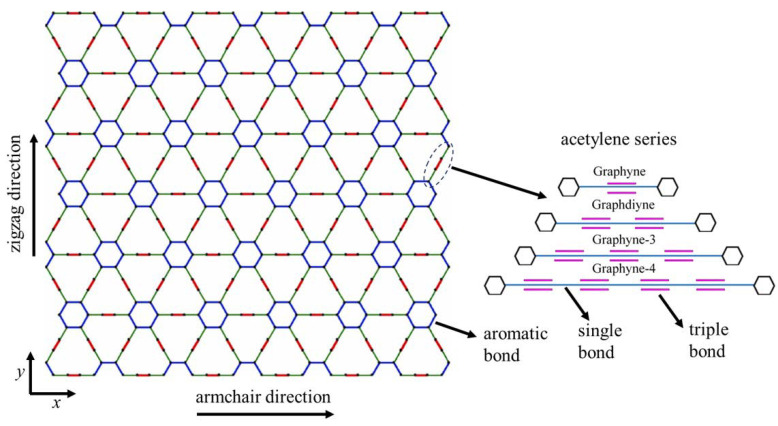
Structural configuration of graphyne.

Graphyne consists of three types of bonds: aromatic, single, and triple. The repeatable unit cell of the structure is the single-triple-single bond interconnection. The number of the unit-cell triple bonds defines the graphyne variation, i.e., one bond yields graphyne, two bonds yield graphdiyne, three bonds yield graphyne-3, and four bonds yield graphyne-4. Figure 1 shows the structural configuration of a graphyne sheet along with the bond variations.

**Figure 2:**
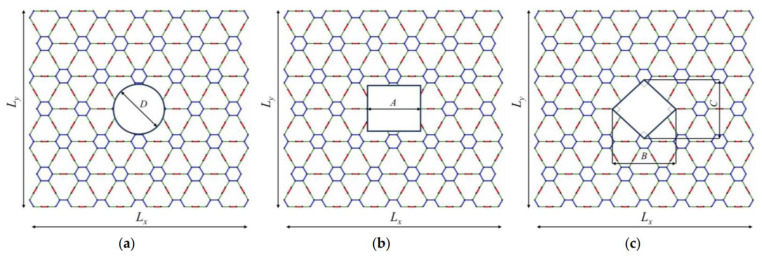
Configuration of graphyne structures with holes: (**a**) circular, (**b**) square, and (**c**) rhombus-type hole.

Figure 2 depicts the geometric failures, which are the circular hole, the square-type hole and rhombus-type hole. The diameter of the circular hole is denoted as *D*, the length of the side of the square-type hole is *A*, and the lengths of the rhombus-type hole are *B* and *C*. In the case studied, the interior angles of the rhombus are 90 degrees. The holes are located at the geometrical center of the structure.

The effects of the geometric parameters of the holes are indirectly calculated via the calculation of the area of the hole. The dimensions of all γ- graphyne models were approximately 10 × 10 nm. The investigations are carried out for five different aspect ratios, including 5%, 10%, 15%, 20% and 25%. The hole aspect ratio *AR* is obtained by dividing the area of the hole (*Af*) by the total area of each nanosheet (*At*).

(1) $$AR=\frac{Af}{At}$$

This approach allows for a more generalized analysis of the impact of hole geometry on the mechanical properties of the graphyne structures, as it accounts for variations in hole size and shape by normalizing the hole area to the total area of the nanosheet.

Building on the aforementioned methodology, the determination of the effect of the parameter becomes straightforward when utilizing the provided formulas for specific hole shapes and given values of AR and At. For a circular hole, the diameter (*D*) can be derived from the equation $D=\sqrt{4ARAt/\pi }$. Similarly, for a square hole, the side length (*A*) is given by $A=\sqrt{ARAt}$. In the case of a rhombus-shaped hole, where both diagonals are equal (*B* = *C*), the diagonal length (*B*) can be calculated using the formula $B=\sqrt{2ARAt}$. These formulas offer a systematic approach to determine the dimensions of the holes based on their shape, ensuring a consistent and accurate analysis. By employing these equations, researchers can effectively gauge the influence of different hole geometries on the mechanical properties of γ-graphyne structures, further enhancing the comprehensiveness of the study.

### 2.2. Force Field Description

The aggregate potential energy of a graphyne molecular arrangement can be depicted as the cumulative energies from interatomic interactions. Considering that nonbonded interactions are negligible for the molecular systems under investigation, and only in-plane attributes are being assessed, the potential energy of a γ-graphyne configuration is articulated as the cumulative energies of bond stretching and bond angle bending:

(2) $$U_{\mathrm{tot}}=\sum U_{r}^{i}+\sum U_{\theta }^{i},$$

where $U_{r}^{i}$ represents the bond stretching term and $U_{\theta }^{i}$ denotes the bond angle bending term, respectively. The parameter *i* denotes the kind of bond, assuming the values *s*, *a*, and *t* correspond to the single, aromatic, and triple bond, respectively. According to the Morse potential field [31], the potential energy terms can be written as

(3) $$U_{r}^{i}=D_{e}^{i}\left\{\left[1-e^{-{Β}^{i}\left(r^{i}-r_{0}^{i}\right)}\right){]}^{2}-1\right\}$$

(4) $$U_{\theta }^{i,j}=\frac{1}{2}k_{\theta }^{i,j}{\left(\theta^{i,\beta }-\theta_{0}^{i,j}\right)}^{2}[1+k_{\mathrm{sextic}}^{i,j}\left(\theta^{i,j}-\theta_{0}^{i,j}{)}^{4}\right]$$

In Equation (2), $r^{i}$ and $r_{0}^{i}$ represent the changed and original bond lengths, respectively. $D_{e}^{i}$ and ${Β}^{i}$ are constants that vary based on the type of bond (*s*, *a*, *t*). In Equation (3), $\theta^{i,j}$ and $\theta_{0}^{i,j}$ are the angles between the deformed and original state of two adjacent bonds *i* and *j*, respectively. $k_{\theta }^{i,j}\;$ and $k_{\mathrm{sextic}}^{i,j}$ are constants that control the linear and nonlinear force-deformation relationship of bond angle bending interactions. It is important to note that nonbonded interatomic interactions and resulting nonlocality typically have minimal impact on the overall mechanical behavior of graphene-like nanostructures and can be omitted in the mathematical formulation.

These formulas can be differentiated to mimic the force field inside the nanostructure using a mix of translational and rotational springs. The longitudinal rigidity of the translational springs is determined by differentiating Equation (2) concerning the variation in bond length ($\Delta r^{i}=r^{i}-r_{0}^{i}$), and the necessary rigidity of the rotational spring can be obtained by differentiating Equation (3) concerning the variation in the bending angle ($\Delta \theta^{i,j}=\theta^{i,j}-\theta_{0}^{i,j}$),

(5) $$F_{r}^{i}\left(\Delta r^{i}\right)=\frac{\partial U_{r}^{i}}{\partial \left(\Delta r^{i}\right)}=2B^{i}D_{e}^{i}\left(1-e^{-B^{i}\Delta r^{i}}\right)e^{-B^{i}\Delta r^{i}},$$

(6) $$M_{\theta }^{i,j}\left(\Delta \theta^{i,j}\right)=\frac{\partial U_{\theta }^{i,j}}{\partial {\left(\Delta \theta^{i,j}\right)}^{2}}=k_{\theta }^{i,j}\Delta \theta^{i,j}\left[1+4\overset{\approx 0}{\overbrace{k_{\mathrm{sextic}}^{i,j}{\left(\Delta \theta^{i,j}\right)}^{3}}}\right]\approx k_{\theta }^{i,j}\Delta \theta^{i,j},$$

where the developed axial force between two bonded atoms $F_{r}^{i}\left(\Delta r^{i}\right)$ is a result of a change in their interatomic distance, while a bending moment $M_{\theta }^{i,j}\left(\Delta \theta^{i,j}\right)$ arises from a change in the angle between two linked bonds.

### 2.3. Finite Element Model

The simulation of potential energies resulting from bond length and bending angle variations is consistent with the finite element formulation outlined in [30,32]. This is achieved by utilizing two nodes, spring-based line elements that connect two bonded atoms. The longitudinal rigidity of the translational springs is determined by differentiating Equation (4) concerning the variation in bond length, while the rotational spring is derived by differentiating Equation (5) with respect to bending angle variation.

(7) $$\frac{\partial F_{r}^{i}}{\partial \left(\Delta r^{i}\right)}=2B^{i}D_{e}^{i}\left(2-e^{-B^{i}\Delta r^{i}}\right)e^{-2B^{i}\Delta r^{i}},\;\frac{\partial M_{\theta }^{i,j}}{\partial {\left(\Delta \theta^{i,j}\right)}^{2}}\approx k_{\theta }^{i,j}.$$

The stiffness matrix of the finite elements and their force-displacement behavior are deduced by using a local Cartesian coordinate system ($\bar{x},\bar{y}$). This results in a 2 × 2 elemental matrix that incorporates two fundamental coefficients in the $\bar{x}$ and $\bar{y}$ directions:

(8) $$k^{\mathrm{el}}=\left[\begin{array}{cccc} k_{\bar{x}}^{\mathrm{el}} & 0 & -k_{\bar{x}}^{\mathrm{el}} & 0\\ 0 & k_{\bar{y}}^{\mathrm{el}} & 0 & -k_{\bar{y}}^{\mathrm{el}}\\ -k_{\bar{x}}^{\mathrm{el}} & 0 & k_{\bar{x}}^{\mathrm{el}} & 0\\ 0 & -k_{\bar{y}}^{\mathrm{el}} & 0 & k_{\bar{y}}^{\mathrm{el}} \end{array}\right].$$

where the symbolization el of the element receives distinct string values: sas, ast, sts, and tst, indicating that the current formulation necessitates the implementation of four kinds of spring-like elements with varying longitudinal and transverse stiffness coefficients, as illustrated in [30,32]. For example, the notation ‘sts’ corresponds to a spring-based element used to describe a triple bond located between two single bonds. The nonlinear axial stiffness coefficients of these elements can be determined using the subsequent equation.

(9) $$k_{\bar{x}}^{\mathrm{el}}=\left\{\begin{array}{c} \;2{\left(B^{s}\right)}^{2}D_{e}^{s}(2-e^{B^{s}\Delta \bar{x}})e^{-2B^{s}\Delta \bar{x}},\;\mathrm{el}=\mathrm{ast},\mathrm{tst}\\ 2{\left(B^{a}\right)}^{2}D_{e}^{a}(2-e^{B^{a}\Delta \bar{x}})e^{-2B^{a}\Delta \bar{x}},\;\mathrm{el}=\mathrm{sas}\\ 2{\left(B^{t}\right)}^{2}D_{e}^{t}(2-e^{B^{t}\Delta \bar{x}})e^{-2B^{t}\Delta \bar{x}},\;\mathrm{el}=\mathrm{sts} \end{array}\right.,$$

(10) $$k^{\mathrm{el}}=\left[\begin{array}{cccc} k_{\bar{x}}^{\mathrm{el}} & 0 & -k_{\bar{x}}^{\mathrm{el}} & 0\\ 0 & k_{\bar{y}}^{\mathrm{el}} & 0 & -k_{\bar{y}}^{\mathrm{el}}\\ -k_{\bar{x}}^{\mathrm{el}} & 0 & k_{\bar{x}}^{\mathrm{el}} & 0\\ 0 & -k_{\bar{y}}^{\mathrm{el}} & 0 & k_{\bar{y}}^{\mathrm{el}} \end{array}\right].$$

The notation el of the element is assigned one of four different string values: sas, ast, sts, and tst. This indicates that the current formulation necessitates the implementation of four types of spring-like elements, each with distinct longitudinal and transverse stiffness coefficients, as outlined in [21,31]. For example, the notation sts represents a spring-like element used to describe a triple bond between two single bonds. The nonlinear axial stiffness coefficients of these elements can be determined using the following equation.

(11) $$k_{\bar{x}}^{\mathrm{el}}=\left\{\begin{array}{c} \;2{\left(B^{s}\right)}^{2}D_{e}^{s}(2-e^{B^{s}\Delta \bar{x}})e^{-2B^{s}\Delta \bar{x}},\;\mathrm{el}=\mathrm{ast},\mathrm{tst}\\ 2{\left(B^{a}\right)}^{2}D_{e}^{a}(2-e^{B^{a}\Delta \bar{x}})e^{-2B^{a}\Delta \bar{x}},\;\mathrm{el}=\mathrm{sas}\\ 2{\left(B^{t}\right)}^{2}D_{e}^{t}(2-e^{B^{t}\Delta \bar{x}})e^{-2B^{t}\Delta \bar{x}},\;\mathrm{el}=\mathrm{sts} \end{array}\right..$$

In accordance with the simplified modeling technique, detailed in [42], the bond angle bending variation can be effectively addressed by utilizing an suitable rigidity coefficient in the $\bar{y}$-direction for each element el=sas,ast,sts,tst. This is provided by:

(12) $$k_{\bar{x}}^{\mathrm{el}}=\left\{\begin{array}{c} \;2{\left(B^{s}\right)}^{2}D_{e}^{s}(2-e^{B^{s}\Delta \bar{x}})e^{-2B^{s}\Delta \bar{x}},\;\mathrm{el}=\mathrm{ast},\mathrm{tst}\\ 2{\left(B^{a}\right)}^{2}D_{e}^{a}(2-e^{B^{a}\Delta \bar{x}})e^{-2B^{a}\Delta \bar{x}},\;\mathrm{el}=\mathrm{sas}\\ 2{\left(B^{t}\right)}^{2}D_{e}^{t}(2-e^{B^{t}\Delta \bar{x}})e^{-2B^{t}\Delta \bar{x}},\;\mathrm{el}=\mathrm{sts} \end{array}\right..$$

To investigate the elastoplastic behavior of a nanostructure, the equilibrium equation for each nonlinear spring-based element can be written as:

(13) $$k^{\mathrm{el}}u^{\mathrm{el}}=f^{\mathrm{el}}.$$

The equilibrium equation is for each hypothetical nonlinear spring-based element, where $u^{\mathrm{el}}$ and $f^{\mathrm{el}}$ represent the respective force and displacement vectors for each element. These vectors have the following notation if i and j are the two nodes of the finite element:

(14) $$u^{\mathrm{el}}={\left[\begin{array}{cccc} u_{\bar{x}i}^{\mathrm{el}} & u_{\bar{y}i}^{\mathrm{el}} & u_{\bar{x}j}^{\mathrm{el}} & u_{\bar{y}j}^{\mathrm{el}} \end{array}\right]}^{T},$$

(15) $$f^{\mathrm{el}}={\left[\begin{array}{cccc} f_{\bar{x}i}^{\mathrm{el}} & f_{\bar{y}i}^{\mathrm{el}} & f_{\bar{x}j}^{\mathrm{el}} & f_{\bar{y}j}^{\mathrm{el}} \end{array}\right]}^{T}.$$

The system of nonlinear equations can be assembled into its final form, through the transformation of the global coordinate system to the elemental stiffness equation for each finite element, in accordance with the constraints of nodal connectivity:

(16) $$K^{\mathrm{el}}(U^{\mathrm{el}})\;U^{\mathrm{el}}=F^{\mathrm{el}},$$

where $F^{\mathrm{el}}$, $U^{\mathrm{el}}$, and $K^{\mathrm{el}}{\left(U\right)}^{\mathrm{el}}$ are the assembled force vector, assembled displacement vector, and assembled deformation-dependent stiffness matrix, respectively.

To simulate the nonlinear mechanical tensile tests, appropriate supports and loads are applied as boundary conditions. The next step is to use an established incremental-iterative method based on the Newton–Raphson algorithm to address the nonlinearity of the global stiffness matrix numerically and determine the response of the structure as a result.

## 3. Results and Discussion

The size of the structure and the number of aromatic rings are indeed inversely related when considering the length of the acetylenic linkage in graphyne structures. Specifically, graphyne, with the shortest acetylenic linkage, accommodates more aromatic rings within a given size compared to its counterparts. As we progress to graphdiyne, graphyne-3, and graphyne-4, the length of the acetylenic linkage increases, which results in a reduced number of aromatic rings for structures of the same size (Figure 1). This relationship has significant implications for the mechanical properties of these materials. Aromatic rings inherently contribute to the rigidity and stability of the structure due to their conjugated and electron-delocalized nature. Therefore, materials with a higher number of aromatic rings, like graphyne, tend to exhibit superior mechanical properties [30,31]. In essence, the more aromatic rings present in the structure, the better the mechanical strength and stability.

The proposed finite element model has been previously validated through comparisons with other available results in the open literature [31] regarding pristine graphyne structures. However, a number of comparisons were performed between the outcomes of this computational approach and related data taken from various literature sources, as provided in Table 1, in order to validate the numerical calculation of the key mechanical properties of γ-graphynes. This comparison serves as further verification of the validity and accuracy of the proposed model. These sources employed different approaches to extract the data. The results indicate a reasonable agreement with the findings of previous studies and demonstrate that the model can be used to predict the mechanical properties of graphyne structures with holes with a high degree of accuracy, providing confidence in the accuracy of the computational scheme, and thus can be useful for the design and optimization of graphyne-based materials and devices.

**Figure 3:**
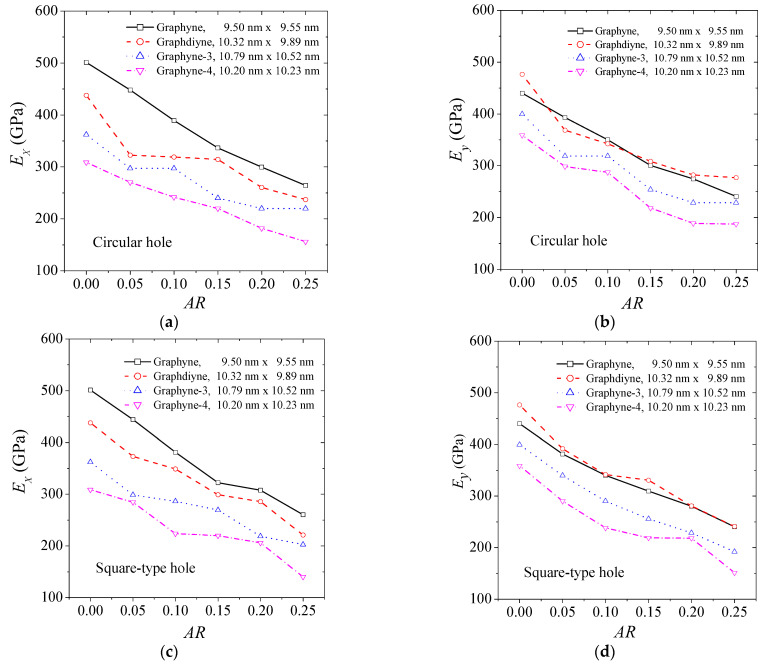

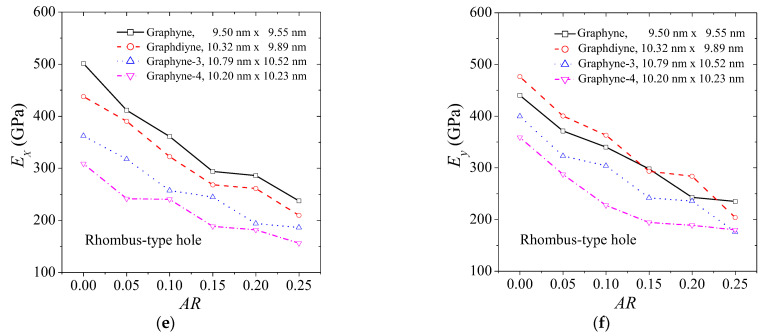
The elastic modulus of graphyne structures vs. aspect ratio in (**a**) x—direction for the circular hole, (**b**) y—direction for the circular hole, (**c**) x—direction for the square hole, (**d**) y—direction for the square hole, (**e**) x—direction for the rhombus-type hole, and (**f**) y—direction for the rhombus-type hole.

The behavior of the elastic modulus of graphyne structures (graphyne, graphdiyne, graphyne-3, graphyne-4) with increasing aspect ratio of the hole is an important aspect of understanding the mechanical properties of these materials. Figure 3 provides insights on this behavior, illustrating the change in elastic modulus for different types of holes. Figure 3a,b show the behavior of the elastic modulus in the *x* and *y* axis for the case of a central circular hole for the four structures. Figure 3b,c depict the case of a square hole, while Figure 3c,d describe the case of a rhombus-type hole. The elastic modulus is seen to decrease as the size of the hole increases in all circumstances. Additionally, the rate of decrease in the elastic modulus with an increase in hole size is similar for all cases. This suggests that the graphyne structures have a relatively consistent response to the introduction of holes in their structure. However, small fluctuations in the elastic modulus may be attributed to the non-symmetric geometric effects caused by the hole creation, depending on the number of atoms and bonds cut off. As the length of the acetylene series increases, the elastic modulus is generally lower in all cases. This highlights the importance of the number of atoms and bonds in determining the mechanical properties of graphyne structures. Close values are observed concerning the elastic modulus in the *y*-direction between the graphyne and graphdiyne specifically, indicating that these two structures may have similar mechanical properties in certain directions.

**Figure 4:**
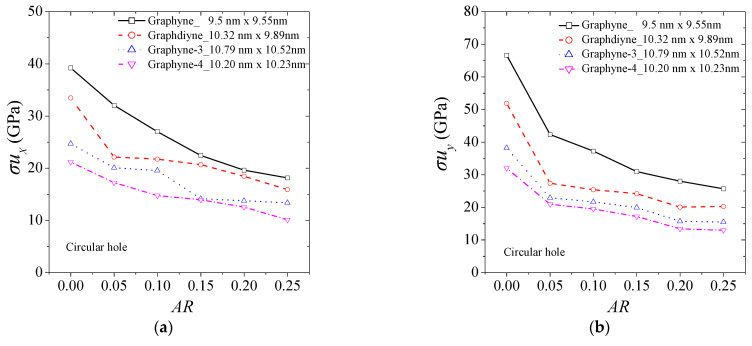

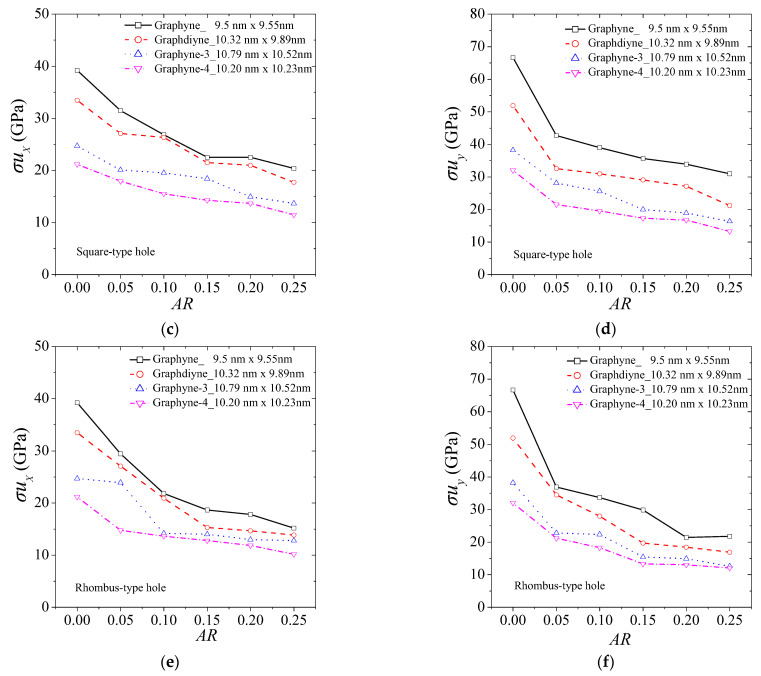
The ultimate tensile strength of graphyne structures vs. aspect ratio in (**a**) x—direction for the circular hole, (**b**) y—direction for the circular hole, (**c**) x—direction for the square hole, (**d**) y—direction for the square hole, (**e**) x—direction for the rhombus-type hole, and (**f**) y—direction for the rhombus-type hole.

The mechanical tensile strength of graphyne structures (graphyne, graphdiyne, graphyne-3, graphyne-4) seems to be considerably affected by the aspect ratio of the hole. Figure 4 illustrates the change in ultimate strength for different types of holes. Figure 4a,b show the ultimate strength in the *x* and *y* axis for the case of a central circular hole for the four structures. Figure 4b,c depict the case of a square hole, while Figure 4c,d describe the case of a rhombus-type hole. As the size of the hole increases, the structure can experience a significant reduction in strength, with losses of up to almost 50% observed for aspect ratios near 0.25. The rate of decrease in the ultimate strength with an increase in hole size is similar for all cases. Small fluctuations in the ultimate strength may be observed for the same reasons as previously.

As the length of the acetylene series increases, the ultimate strength is generally lower in all cases. This emphasizes the significance of the number of atoms and bonds in establishing the mechanical properties of graphyne structures.

**Figure 5:**
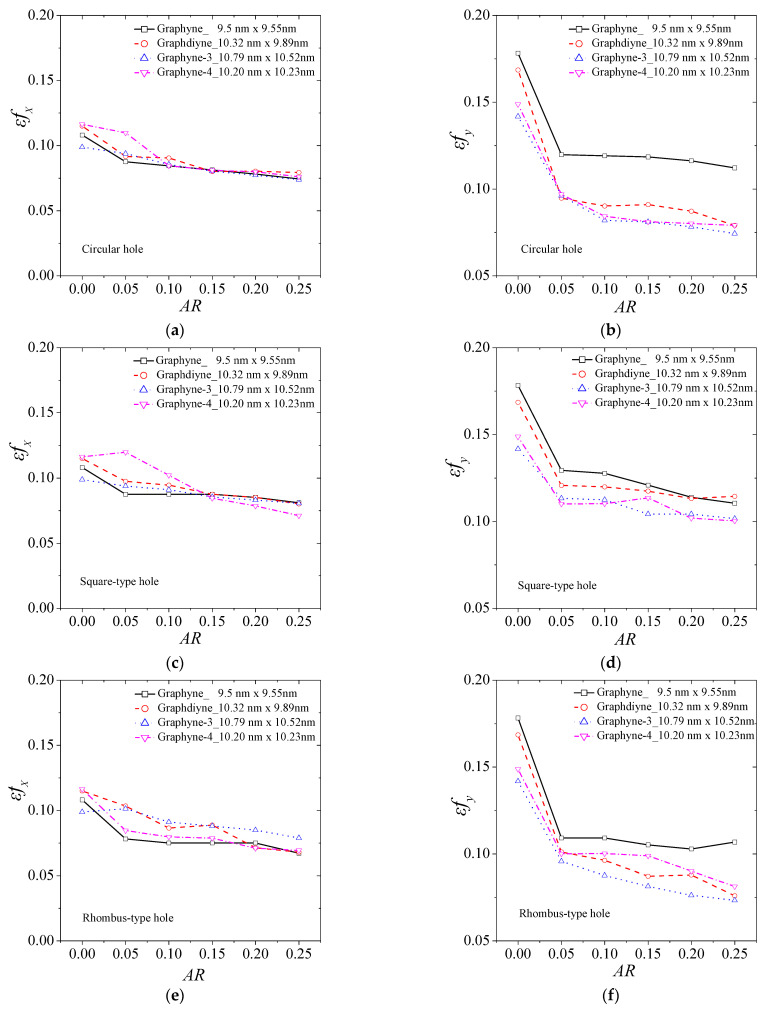
The fracture strain of graphyne structures vs. aspect ratio in (**a**) x—direction for the circular hole, (**b**) y—direction for the circular hole, (**c**) x—direction for the square hole, (**d**) y—direction for the square hole, (**e**) x—direction for the rhombus-type hole, and (**f**) y—direction for the rhombus-type hole.

In Figure 5, we observe the variation in fracture strain for different types of holes. Figure 5a,b present the fracture strain in the *x* and *y* axis for the case of a central circular hole for the four structures. Figure 5b,c depict the case of a square-type hole, while Figure 5c,d describe the case of a rhombus-type hole. It is noticeable that in all cases, the rate of decrease in the fracture strain with an increase in hole size is similar for all cases. Slight variations in the fracture strain can be attributed to non-symmetric geometric effects caused by the hole creation, depending on the number of atoms and bonds cut off. Similarly, here, as the length of the acetylene series increases, the fracture strain is generally lower in all cases.

We conducted a nonlinear regression analysis to extract analytical equations that capture the hole-dependent variations in mechanical properties of the four types of graphyne with central holes, aiming to enable a more systematic and simplified prediction of their mechanical properties. The following fitting 3-parameter function of hole aspect ratio was used for all of the attempted property approximations:

(17) $$Y/Y_{0}=a+be^{-ARc},\;$$

where *Y* is the suggested mathematical model to fit the data computed for all examined properties *E_x_*, *E_y_*, *σu_x_*, *σu_y_*, *εf_x_*, and *εf_y_* whereas, *a*, *b*, and *c* are constant parameters that must be found using a different regression procedure for each mechanical property variation. *Y_0_* is the corresponding property of pristine graphyne structure. Equation (20) from [31] is a valuable tool for predicting the mechanical properties of graphyne structures, by considering the effect of size variations. This equation can be used to estimate *Y_o_*, an important parameter for understanding the mechanical behavior of these structures. By utilizing Equation (17), which relates *Y_o_* to the corresponding mechanical properties *Y*, predictions of any size can be made, providing a comprehensive understanding of the mechanical response of graphyne structures with holes. 

**Figure A1:**
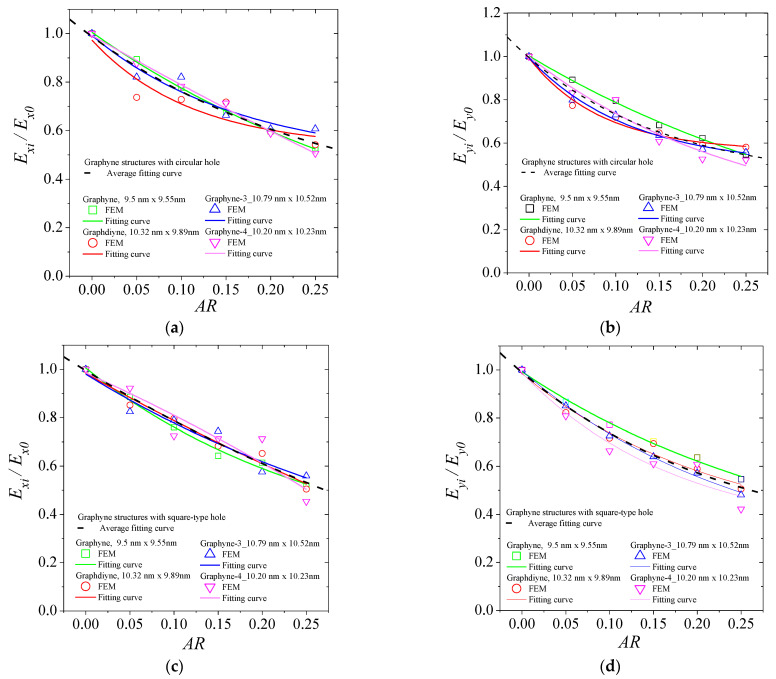

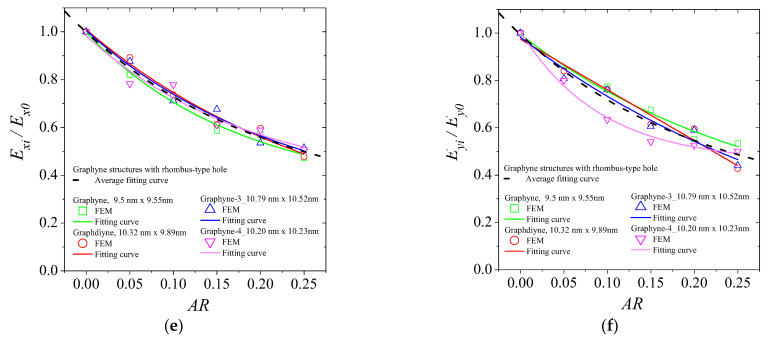
Fitting curves for the normalized Young’s modulus of graphyne structures vs. aspect ratio in (**a**) x—direction for the circular hole, (**b**) y—direction for the circular hole, (**c**) x—direction for the square hole, (**d**) y—direction for the square hole, (**e**) x—direction for the rhombus-type hole, and (**f**) y—direction for the rhombus-type hole.

**Figure A2:**
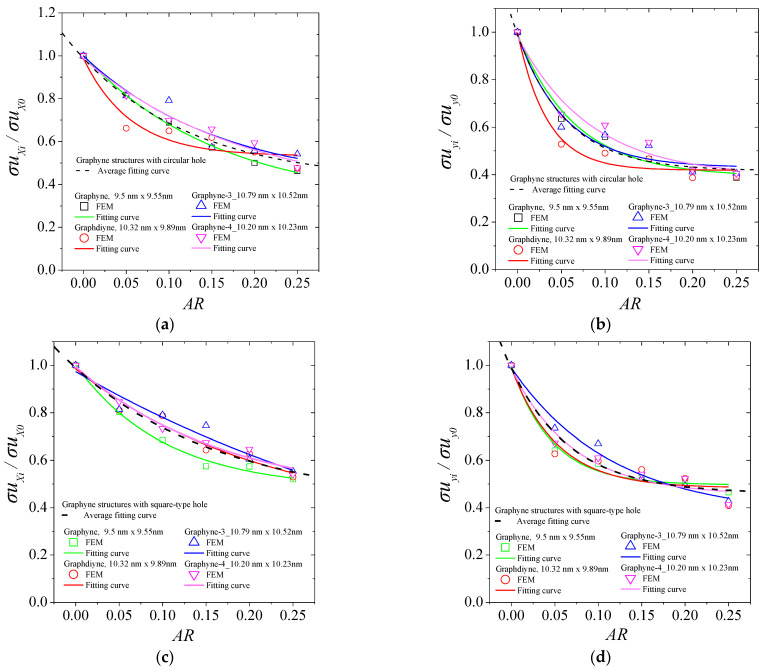

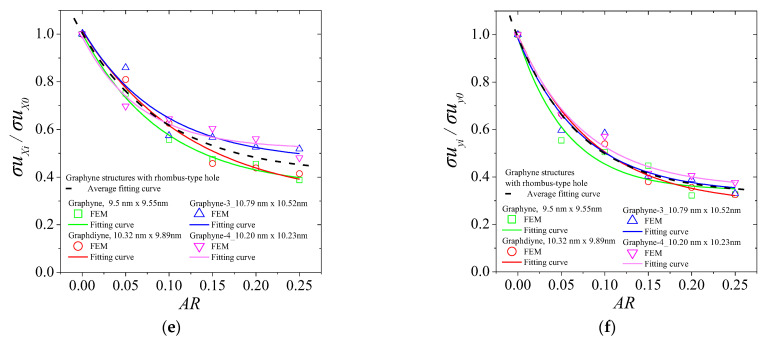
Fitting curves for the normalized ultimate tensile strength of graphyne structures vs. aspect ratio in (**a**) x—direction for the circular hole, (**b**) y—direction for the circular hole, (**c**) x—direction for the square hole, (**d**) y—direction for the square hole, (**e**) x—direction for the rhombus-type hole, and (**f**) y—direction for the rhombus-type hole.

**Figure A3:**
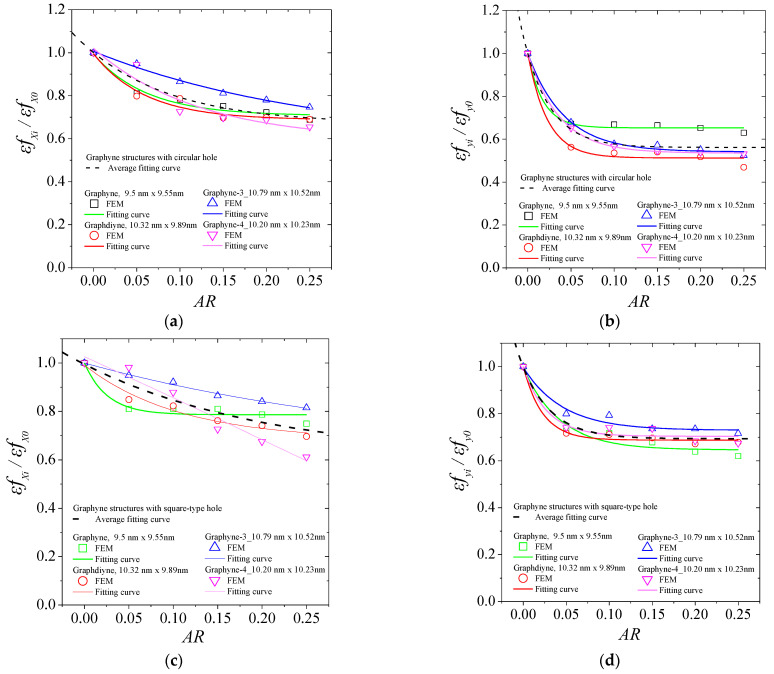

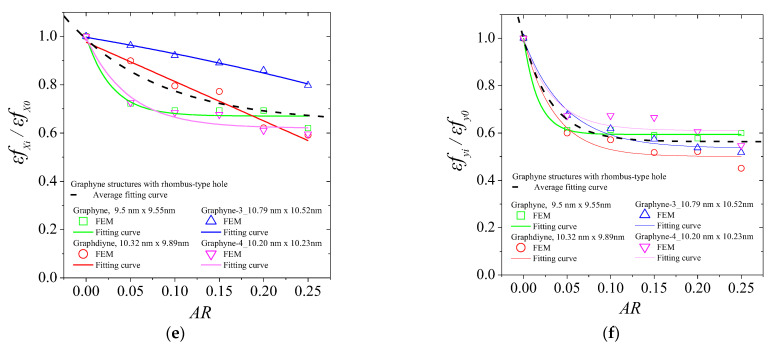
Fitting curves for the normalized fracture strain of graphyne structures vs aspect ratio in (**a**) x—direction for the circular hole, (**b**) y—direction for the circular hole, (**c**) x—direction for the square hole, (**d**) y—direction for the square hole, (**e**) x—direction for the rhombus-type hole, and (**f**) y—direction for the rhombus-type hole.

By incorporating the FEM results and performing a regression analysis using Equation (17), the optimized values of the parameters can be calculated. Appendix A presents the regression curves for each graphyne structure type, hole type, and mechanical property in Figure A1, Figure A2 and Figure A3. These figures also include the average curve obtained by considering a single equation for all graphyne structure types. Table 2 provides the parameter values for the unique equation applied to all graphyne structure types. For a more accurate prediction, Table A1 contains the values of the parameters for Equation (17) that were obtained from the fitting process for mechanical properties data of graphyne and each hole type. The fitting process for the mechanical properties data based on Equation (17) yielded the parameter values shown in Table A2, regarding the graphdiyne. Table A3 and Table A4 provide the parameter values obtained from fitting Equation (17) to the mechanical properties data for each hole type in graphyne-3 and graphyne-4, respectively.

The nonlinear FEA method was implemented under large deformations to examine the mechanical properties of γ-graphyne structures with circular, triangle, and rhombus hole configurations. The study used an atomistic spring-based finite element model, simulating the interatomic interactions between carbon atoms. This approach provided a comprehensive understanding of the mechanical response of graphyne structures with holes, enabling a more systematic and simplified prediction of their mechanical properties. In addition to the FEA used in this study, the nonlinear response of γ-graphyne structures can also be investigated using several other robust numerical modeling methods. For instance, the finite difference method [46], Bezier multi-step method [47], and differential quadrature method [48] can provide alternative approaches to understanding the nonlinear response of γ-graphyne structures and can be considered for future studies or comparisons.

The study methodically investigates the nonlinear mechanical response of γ-graphyne structures, including variations such as graphyne, graphdiyne, graphyne-3, and graphyne-4, under the influence of different hole geometries (circular, square, and rhombus) and aspect ratios (5%, 10%, 15%, 20%, 25%). The investigation reveals that hole geometry significantly impacts the elastic behavior, deformation, and strength of the structures, with the elastic modulus, ultimate strength, and fracture strain decreasing as hole size increases. This is attributed to the variations in stress distribution and deformation patterns caused by the holes. The study also presents analytical equations derived from regression analysis to capture hole-dependent variations in mechanical properties, providing a valuable tool for systematic and simplified prediction of mechanical properties. This comprehensive analysis underscores the importance of considering hole geometries in the design and optimization of graphyne-based materials and devices, ultimately aiding in the development of materials with desired mechanical properties for specific applications.

The study of hole geometry, encompassing circular, triangular, and rhombus configurations, is pivotal for comprehending the nonlinear mechanical behavior of γ-graphyne structures. Different hole geometries lead to variations in the stress distribution, deformation patterns, and ultimately, the mechanical properties of the material. Specifically, the hole geometry influences the initiation and propagation of cracks, affecting the material’s strength and failure mechanisms. This understanding is crucial for optimizing the design and fabrication of γ-graphyne structures, as it can lead to enhanced mechanical properties, making the material more suitable for various applications, such as in nanoelectronics and nanomechanical systems.

## 4. Conclusions

In this study, we investigated the effect of hole geometry on the mechanical nonlinear response of γ-graphyne structures, including graphyne, graphdiyne, graphyne-3, and graphyne-4 structures, under large deformations using a finite element analysis (FEA) method. We found that the size and shape of the holes significantly impacted the structures’ elastic behavior, deformation, and strength, highlighting the importance of considering hole geometries in the design and fabrication of graphyne-based materials and devices. The results indicated that the size and shape of the holes significantly impacted the structures’ elastic behavior, deformation, and strength. Specifically, the elastic modulus of the graphyne structures decreased with an increase in the size of the hole, and the ultimate strength and fracture strain experienced a similar rate of decrease with an increase in hole size. Small fluctuations in these properties were observed due to non-symmetric geometric effects caused by the hole creation. Our study provides a valuable tool for predicting the mechanical properties of graphyne structures with holes by incorporating the effect of size variations, enabling a more systematic and simplified prediction of their mechanical properties. The analytical equations extracted from the regression analysis, which capture the hole-dependent variations in mechanical properties, were presented. This study highlights the importance of considering the hole geometries in the design and fabrication of these materials, providing a comprehensive understanding of the mechanical response of graphyne structures with holes, which can be useful for the design and optimization of graphyne-based materials and devices. Overall, our findings can aid in the optimization of the design of graphyne structures for specific mechanical applications.

## Figures and Tables

**Table 1:** Comparison of the mechanical characteristics of pristine graphene predicted by the finite element method with equivalent findings from previous investigations.

	Method	*L_x_* × *L_y_* (nm × nm)	*E_x_* (GPa)	*E_y_* (GPa)	*σu_x_* (GPa)	*σu_y_* (GPa)	*εf_x_*	*εf_y_*
Graphyne	FEM (present)	9.5 × 9.55	501.2	440.1	39.2	66.6	0.108	0.178
	FEM [31]	10.9 × 9.8	512.6	507.8	48.6	72	0.13	0.17
	FEM [43]	9.5 × 9.55	481.6	419.0	-	-	-	-
	MD [5]	10 × 10	-	-	45	63.96	0.11	0.177
Graph- diyne	FEM (present)	10.32 × 9.89	439.2	476.4	33.4	51.8	0.115	0.168
	FEM [3]	10.7 × 10.1	360.6	383.6	-	-	-	-
	MD [44]	10 × 10	312.5	270.3	29.8	65.1	0.109	0.208
	DFT [45]	7 × 7	-	384.8	-	-	-	-
Graphyne-3	FEM (present)	10.8 × 10.5	364.9	399.5	24.8	38.2	0.096	0.141
	FEM [43]	11.2 × 10.7	288.9	307.8	-	-	-	-
	MD [5]	10 × 10	243.1	212.3	22.8	65.3	0.109	0.223
Graphene-4	FEM (present)	10.2 × 10.23	310.2	345.8	21.15	32.5	0.116	0.166
	FEM [31]	10.8 × 10.5	364.9	399.5	24.8	38.2	0.096	0.141
	FEM [43]	10.6 × 10.4	239.7	257.4	-	-	-	-
	MD [44]	10 × 10	199.5	168.3	18.4	65.3	0.108	0.224

**Table 2:** Parameter values of Equation (17) for the analytical prediction of mechanical properties.

		*E_x_*	*E_y_*	*σu_x_*	*σu_y_*	*εf_x_*	*εf_y_*
Circular hole	*a*	0.2655	0.41855	0.41263	0.41566	0.66951	0.56134
	*b*	0.72223	0.57663	0.57642	0.57436	0.33157	0.4381
	*c*	0.26581	0.16479	0.13383	0.05611	0.10066	0.03045
	*R^2^*	0.94	0.95	0.93	0.95	0.84	0.91
Square hole	*a*	−0.40512	0.25461	0.41804	0.45973	0.60684	0.69359
	*b*	1.39502	0.73541	0.57141	0.52862	0.38811	0.30464
	*c*	0.62309	0.23804	0.17159	0.06837	0.20698	0.03318
	*R^2^*	0.94	0.95	0.94	0.95	0.77	0.91
Rhombic hole	*a*	0.26796	0.26272	0.40943	0.32775	0.64213	0.56321
	*b*	0.73041	0.73113	0.59529	0.66189	0.34733	0.43487
	*c*	0.21701	0.21103	0.09505	0.07381	0.10297	0.0326
	*R^2^*	0.97	0.95	0.94	0.97	0.78	0.92

## Data Availability

Data supporting the findings of this study are available on request from the corresponding author.

## Appendix A

Figure A1 shows the fitting curves based on Equation (17) that fit the FEM data for each structure and hole type, as well as the corresponding average fitting curve, which represents the effect of hole magnitude on the Young’s modulus of the graphyne structures. Figure A2 displays the fitting curves for the ultimate tensile strength, while Figure A3 presents the corresponding fitting curves for the tensile fracture strain.

Table A1, Table A2 and Table A3 provide the values of the parameters obtained from the fitting process for Equation (17) applied to the normalized Young’s modulus, ultimate tensile strength, and tensile fracture strain data, respectively.

The presented tables, namely Table A1, Table A2, Table A3 and Table A4, provide crucial parameter values for Equation (17), which is an analytical model used to predict the mechanical properties of different types of graphyne materials with circular, square, and rhombic holes. Specifically, Table A1, Table A2, Table A3 and Table A4 contain the parameter values for graphyne, graphdiyne, graphyne-3, and graphyne-4, respectively. These tables provide valuable information for designing and engineering materials with specific mechanical properties by controlling the hole shape and the material type. The *R*^2^ values in the tables indicate the goodness of fit of the analytical model and can be used to evaluate the reliability of the predicted mechanical properties. Overall, these tables offer useful insights into the mechanical properties of graphyne materials with different hole shapes, and their practical applications in material science and engineering can be significant.

**Table A1:** Parameter values of Equation (17) for the analytical prediction of mechanical properties of graphyne.

		*E_x_*	*E_y_*	*σu_x_*	*σu_y_*	*εf_x_*	*εf_y_*
Circular hole	*a*	−0.01328	−0.02175	0.31215	0.39201	0.70679	0.65288
	*b*	1.01861	1.02460	0.68999	0.59942	0.28739	0.34707
	*c*	0.39087	0.42385	0.15879	0.06569	0.06158	0.01771
	*R^2^*	0.99000	0.99000	0.99000	0.98000	0.95000	0.98000
Square hole	*a*	0.19684	0.15620	0.47900	0.49592	0.78609	0.64616
	*b*	0.80854	0.83635	0.52249	0.49939	0.21336	0.34956
	*c*	0.27754	0.34094	0.10267	0.04676	0.02598	0.04227
	*R^2^*	0.98	0.99	0.99	0.97	0.90	0.94
Rhombic hole	*a*	0.32312	0.18802	0.35359	0.34209	0.67028	0.59384
	*b*	0.6737	0.80686	0.65038	0.64546	0.32904	0.40612
	*c*	0.17649	0.28148	0.09313	0.05726	0.02908	0.01662
	*R^2^*	0.98	0.97	0.99	0.94	0.93	0.99

**Table A2:** Parameter values of Equation (17) for the analytical prediction of mechanical properties of graphdiyne.

		*E_x_*	*E_y_*	*σu_x_*	*σu_y_*	*εf_x_*	*εf_y_*
Circular hole	*a*	0.52877	0.56265	0.53169	0.41842	0.68781	0.51250
	*b*	0.44434	0.43016	0.45601	0.57879	0.30756	0.48722
	*c*	0.11159	0.08445	0.05557	0.03396	0.06053	0.02279
	*R^2^*	0.82000	0.98000	0.87000	0.96000	0.94000	0.97000
Square hole	*a*	−1.22782	0.30336	0.34185	0.48296	0.68108	0.68787
	*b*	2.21081	0.68163	0.64310	0.50637	0.31021	0.31189
	*c*	1.08266	0.22266	0.21818	0.05221	0.10768	0.02238
	*R^2^*	0.96	0.96	0.94	0.89	0.95	0.98
Rhombic hole	*a*	0.11455	61,266	0.29567	0.28294	−96,147	0.50061
	*b*	0.89455	−61,265	0.7189	0.71424	96,148	0.49665
	*c*	0.28715	−28,643	0.12369	0.08568	58,937	0.03542
	*R^2^*	0.97	0.95	0.97	0.99	0.9	0.96

**Table A3:** Parameter values of Equation (17) for the analytical prediction of mechanical properties of graphyne-3.

		*E_x_*	*E_y_*	*σu_x_*	*σu_y_*	*εf_x_*	*εf_y_*
Circular hole	*a*	0.47251	0.49490	0.39114	0.42952	0.58107	0.54080
	*b*	0.51969	0.49912	0.60804	0.55934	0.42564	0.45870
	*c*	0.16870	0.11669	0.16182	0.05451	0.26025	0.04193
	*R^2^*	0.91	0.98	0.90	0.93	0.98	0.99
Square hole	*a*	0.01813	0.18307	−0.03830	0.36855	0.61464	0.72907
	*b*	0.96220	0.81386	1.01187	0.62103	0.38556	0.26762
	*c*	0.42336	0.25694	0.47241	0.11561	0.37605	0.04605
	*R^2^*	0.91	0.99	0.91	0.97	0.98	0.95
Rhombic hole	*a*	0.24141	−0.0613	0.46377	0.33207	1.38875	0.53561
	*b*	0.76417	1.04379	0.5563	0.65178	−0.39214	0.46029
	*c*	0.23271	0.36545	0.09018	0.07463	−0.62357	0.04771
	*R^2^*	0.97	0.96	0.9	0.93	0.98	0.98

**Table A4:** Parameter values of Equation (17) for the analytical prediction of mechanical properties of graphyne-4.

		*E_x_*	*E_y_*	*σu_x_*	*σu_y_*	*εf_x_*	*εf_y_*
Circular hole	*a*	−2.31049	0.20093	0.32807	0.39183	0.58986	0.53511
	*b*	3.30293	0.79905	0.65845	0.59124	0.43111	0.46471
	*c*	1.57087	0.25039	0.19239	0.08284	0.12130	0.03638
	*R^2^*	0.99000	0.93000	0.96000	0.94000	0.87000	0.99000
Square hole	*a*	2.34985	0.30658	0.43378	0.45300	14.08941	0.70433
	*b*	−1.36430	0.68101	0.56028	0.53464	−13.06307	0.29473
	*c*	−0.82667	0.17919	0.17312	0.07094	−7.71899	0.02722
	*R^2^*	0.82	0.91	0.97	0.93	0.94	0.93
Rhombic hole	*a*	0.34482	0.45448	0.51764	0.35273	0.62012	0.61046
	*b*	0.63847	0.55286	0.47121	0.64212	0.37527	0.38635
	*c*	0.19142	0.09276	0.06646	0.07679	0.04731	0.03378
	*R^2^*	0.92	0.99	0.93	0.98	0.94	0.88
